# *Should I stay or should I go?* Why nurses are leaving community nursing in the UK

**DOI:** 10.1186/s12913-023-09163-7

**Published:** 2023-02-16

**Authors:** Michaela Senek, Steve Robertson, Rachel King, Emily Wood, Tony Ryan

**Affiliations:** Division of Nursing & Midwifery, Department of Health Sciences, University of Sheffield, Sheffield, UK

**Keywords:** Community nursing, Intention-to-leave, Staffing, Working conditions, Overtime, Missed care

## Abstract

Worsening working conditions for nursing workforce has seen a massive exodus of staff, particularly in community nursing in the UK. Aim: The study aim was to map working conditions as well as identify differentiating characteristics of community nurses that intend to leave their profession. Design: Eligibility criteria were community nurses working in all 4 UK countries. All data was collected by means of a cross-sectional survey via the largest closed, private community nursing online-forum. Logistic regression was carried out to ascertain the effects of the variables on the intention to leave. Results: The total number of respondents was 533. Findings showed that one in two of all community nurses (≈46%) are reporting job dissatisfaction. Length of unpaid overtime per shift (odds increase by 30% for each hour of overtime), manager support, proportion of permanent staff, team size, shift length, travel mileage, worsened conditions in the last year and overall self-rated working conditions were differentiating factors between those that intended to leave the job. The proportion of permanent staff on the team and perceived lack of support from management best predicted the likelihood of leave rates. Our findings imply that low nurse retention will fuel an even higher exodus because job dissatisfaction is highest on teams with lowest permanent staff ratios. Poor management that is inept at supporting frontline staff means that the fundamental retention issues are exacerbated and will not stop the unprecedented crisis that is predicted to lead to a collapse of care provision in community settings. Nurses play a central role and are ‘key’ to delivering the much- desired patient-centred care’ therefore their well-being and job satisfaction should become a priority for policymakers.

- Lack of resources is crippling community nursing services in the UK
- Removal of nursing bursary and financial cuts have caused workforce demoralisation in an already understaffed health care setting.
- Workforce pressures are contributing to mass exodus of community nurses

What the paper adds

- Majority of respondents (≈90%) reported that they did not have a full complement of permanent staff on their last shift. Out of these, 1 in 2 respondents reported that they had missed care despite overtime hours (78% carried out unpaid overtime on their last shift).
- Amount of unpaid overtime work, lack of management support, proportion of permanent staff on shift and self-rated overall working conditions are shown to be significant predictors of nurses’ Intention to Leave.
- The odds of intending to leave increases by 30% for every hour of unpaid overtime reported
- Respondents who felt supported by their managers have 83.9% lower odds of reporting intention to leave.
- Reducing understaffing by 20% will result in a 34% drop in the odds ratio of a nurse reporting intention to leave.

## Introduction

A well-functioning community care was listed as one of the nine pillars of a good health system by the European region World Health Organisation (WHO) … and best value for money’ (p.4–5), [1, 2]. Even prior to the COVID-19 pandemic, several European policy think tanks identified the importance of coordinated primary care reform policies and workforce policies. Their findings showed that nations in which the two policies misaligned were not as good at combating non-communicable diseases and other public health challenges [3]. The adjustment of the health services across Europe during the COVID-19 pandemic has demonstrated the pivotal role of primary care. An exploratory study by Wanat et al., during the pandemic in eight European countries found the responsiveness and proper resourcing of primary care to be critical during an infectious disease crisis [4]. Recommendations by the WHO that followed were to strengthen primary care services in their combat against the COVID-19 pandemic [5]. However, despite these recommendations most nations’ priorities have been focused on acute settings [6].

In the UK, the role of the community nurse is to carry out practice that is preventative, curative, and rehabilitative [7]. Community nurses are registered with the Nursing and Midwifery Council (NMC), educated to degree level, and provide care across a range of community settings (the home, care homes and clinics). District nurses are more senior. Also registered with the NMC and educated to degree level, they have obtained additional qualifications and tend to lead teams of other staff, playing a vital role in coordinating and orchestrating services and prescribing medication. District and community nurses make up a fraction of the overall UK nursing workforce, approximately. The Specialist district nursing qualification is obtained after a year-long university course. It includes academic course study and practical experiential learning within district nursing teams. It is estimated that between 30 and 50% of community nurses hold a specialist qualification.

Despite policy ambitions announced in 2009–2010, to offer more care close to home, a continued lack of resources, monitoring and oversight have shown a continued undervaluing and lack of appreciation for community services [8]. This has resulted in the number of qualified community nurses in the UK falling sharply by 42% between 2010 and 2018 [9]. This fall in numbers has not been reflected in other sectors of the UK nursing workforce in such dramatic fashion. The continuous underfunding of community care in the UK has created pressures that continue to compromise quality of care [10].

Further government policies, such as removal of nursing bursary, financial cuts, and workforce demoralisation, have seen this particular specialty brought to a state of crisis [11]. This negative development has peaked at a time when the demand on community nursing services has increased significantly, both in terms of the number of patients seen and the complexity of care provided. This pressure has become increasingly unmanageable in the period 2019–2021, during the COVID-19 pandemic, resulting in adverse effects on patient care [12].

The community nursing workforce pressures noted in a UK context are not exceptional. A recent review of the international literature, centred on nursing turnover in the community [13], indicates a range of factors impacting upon job satisfaction and intention to leave. These include remuneration, but also age and length of time in post, managerial style, and workload. The direct impact on the quality of care in community services is difficult to quantify. This is mainly due to a wide range of national indicators, which focus on organisation of care [14]. To date, the largest community nursing primary study in the UK carried out in early 2020 reported high levels of missed care in all categories of nursing care, severe understaffing, and a higher burden on those that are left in the profession (Senek et al., 2022). It is anticipated that high levels of burnout in community nursing is further worsening retention and recruitment of staff [15]. This evidence suggests that community services are stretched beyond breaking point, at a time when they are expected to bear a heavier load due to the pressure on hospital services in the third wave of COVID-19 pandemic. There is evidence that, along with other professionals and whilst remaining within existing scope of practice, community and district nurses have extended their roles and remit during the pandemic allowing general practitioners to see patients with more complex needs that would otherwise need to be looked after in the hospital setting [16]. As a result, the Department of Health estimates that demand for care will rise by a greater level than this staffing increase could match [17].

These worsening working conditions are believed to have a major impact on retention and recruitment of nursing staff. Previous review work on nurses’ intention to leave identified job-related determinants that had impact such as stress and job satisfaction, burnout, and job commitment. Job satisfaction which was shown to be strongly correlated with intention to leave among nursing staff [18] was a result of nurses’ inability to provide the best care that they wanted to (i.e. missed care) [19]. In fact, missed care was shown to be the largest determinant of nurses’ job dissatisfaction, followed by *perceived lack of support*, *no action taken when concerns are raised*, if they could not *take a break*, *unpaid overtime* and if the shift is *understaffed* [19] .

Whilst these individual indicators are important, these are situated within a complex set of relationships, involving the individual and the organisational context. A review of causal models, which included 24 papers assessing models of nursing turnover, identified burnout, job stress, organisational commitment, job satisfaction, organisational culture and empowerment as having directional relationships and with varying degrees of strength [20]. Similarly, a review by Daouk-Öyry et al., presented a conceptual (JOINT) model of turnover where three concepts of turnover - the interpersonal level (managerial style and relationships); the job level (job demands and job control) and the organisational level (human resources practices and structure), were moderated by individual level (demographics, personal characteristics, job attitude, health and well-being) and national level (labour supply and legislation) characteristics on turnover [21]. It then derived a model of interaction and interplay of the concepts that result in nurse turnover. This model was proposed to test hypothesized relationships and overall management practices [22] and provides an important backdrop to this study.

For a while, community nursing moved up the political agenda because of UK government policy commitments to move care closer to home, to address health inequalities and to prevent disease [23]. However, the system has not sufficiently recognised the vital strategic importance of community nursing services in realising a resilient health service. As a result, the health system faces significant challenges of rising demand at a time of constrained resourcing and capacity. This study aimed to examine the real-time working conditions and individual and organisational factors and their association with community nurses’ intention to leave across the UK.

### Study design

We carried out a questionnaire of community nurses in the UK to explore differences in individual and organisational factors between those nurses that intend to leave and those that intend to stay in the community nursing profession. The data was collected by means of a cross-sectional survey via the largest closed, private community nursing online-forum. Logistic regression was carried out to ascertain the effects of the variables on the intention to leave. Data was collected between February 8th to April 26th, 2021. A web link to the questionnaire was posted on a closed and exclusive group forum by the group administrator. Qualtrics© web software was used to administer the questionnaire. Qualtrics© also operate on protected high-end firewall systems and uses encryptions for all transmitted data.

### Survey development

The questionnaire had two components. One section focused on the prevalence and types of missed care and the other section focused on topics relating to working conditions. Both sections of the questionnaire were based on a validated questionnaire of community nurses working conditions by Phelan et al., [24]. This was followed by an iterative process of engagement with lead members of the UK Royal College of Nursing (RCN) to finalise the questionnaire. RCN participants included national professional nursing leaders in the fields of end-of-life care, education and district nursing and a district nursing academic. Iterations of the amended tool were circulated over a 6-week period and a consensus reached on relevance of the final items for inclusion and exclusion. The total number of questions in the questionnaire was 62. The questionnaire was piloted with 6 community nurses. The completion time was between 30 and 45 minutes.

### Recruitment and consent

Participants were recruited via the largest national Community Nursing Forum in the UK with over 6000 members, hosted within a closed, private social media group. Eligibility criteria were; community nurse professions from any of the four countries of the UK (England, Wales, Northern Ireland or Scotland). Participants were required to indicate eligibility by confirming their registered status.

### Participants

Community nurses of all Agenda for Change Band grades (NHS terms and conditions of service for non-medical staff) were eligible to take part. RNs with or without a Specialist District Nurse Practitioner Qualification (SPQDN) qualification were eligible to take part. The Specialist community nursing qualification is obtained after a year-long university course. It includes academic course study and practical experiential learning within community nursing teams. It is estimated that between 30 and 50% of community nurses hold a specialist qualification. We included all community nursing roles in our sample, if the participants themselves identified at community and/or district nurse. This could include any of the community nursing sub-specialities in the UK, for instance public health, occupational health.

For this study, we aimed to achieve a response rate of at least 10%. Out of the approximately 6000 forum members, 859 joined the study and started to complete the questionnaire. This is a response rate of 14%. However, out of these 859 responses, only 533 met the inclusion criteria (i.e., completion rate of 90% + of the questionnaire). Therefore, 326 responses were excluded in the final analysis. Therefore, our final response rate was just below the target at 8.8%.

### Ethical approval

The study was approved by [Blinded for Peer review] ethics committee *(REDACTED).*

### Confidentiality and safeguarding

All data collected during remained confidential and was only accessible to members of the research team. Data was stored on a secure server. All data was strictly anonymized for publication. No participants are identified in the publication.

### Measured outcomes

We collected information that can be categorised into following domains: demographics, frequency, and type of missed care, staffing levels, reasons for missed care, job satisfaction and intention to leave.

### Demographic variables

We collected non-identifiable participant data including role title, whether they hold an SPQDN qualification, age, gender, years in practice, Agenda for Change (Afc) pay grade (where band 5 is typical of those with least experience and/or level of education, bands 6, 7, and 8 are typical of those with higher levels of education/and or experience), part-time/full time working, country of the UK, and first four digits of their work postcode.

### Missed care and types of missed care

‘Due to a lack of time did you leave necessary care (any aspect of required patient care) undone on your last shift? The response options were ‘yes’ or ‘no’.

### Working conditions

Participants listed their current caseload (current active caseload measured as the total number of patients that are assigned to them individually), additional caseload attributable to staff absence on their last shift (additional caseload measured as additional number of patients allocated to them on that shift due to staff absence) and the proportion of their caseload that are COVID-19 related cases (additional COVID caseload measured as number of patients that are have COVID or COVID-related complications), caseload increase due to COVID (Has your caseload increased due to COVID-19 pandemic). We also asked how many patients they had seen on their last shift (measured as total Number of Patients seen). They were also asked about the number of RN vacancies in the team (Proportion of Permanent Staff variable), Total hours worked on the last shift (length of shift measured as hours and minutes), travel mileage on the last shift (travel mileage), manager support (measured as dichotomous reply Yes (supported) No (unsupported), have working conditions worsened in the last year (measured as Yes/No dichotomous reply), and time spent per patient (measured in minutes).

## Data analysis

Staffing ratios, caseloads, additional caseloads, and proportion of vacancies were calculated using mean and standard deviation. Other individual or organisational factors are presented using frequencies and percentages. We report missing cases for each variable.

Chi-squared Tests were carried out on categorical variables with Z-tests used for within group comparisons. For scalar variables a Kruskal-Wallis test was used to test the differences in organisational and individual factors.

To explore the differences in responses between participants that responded yes/no/maybe to the intention to leave the job question, we carried out Kruskall-Wallis for linear variables and Mann-Whitney with Bonferroni Correction was done to check significance. Chi-square analyses was used to analyse the binary variables. Those variables that were significant were included in the Backward LR analysis.

Individual nurse factors and structural/organisational factors and patient factors (nurse report) in missed care were explored more fully. Individual nurse factors included: SPQDN qualification and years of community nursing and intention to leave. The patient factors (nurse report) were current active caseload and additional cases to current caseload on last shift. Organisational factors were length of last shift (hours), overtime on last shift (minutes), total travel mileage on your last shift, sufficient support from manager (‘sufficient’ as individually defined by each respondent as it is based on self-perceived and is self-reported), number of RNs on the team, percentage of staff permanent and full complement of staff on last shift.

Further exploration of the factors found significant in the bivariate analysis was done by means of logistical regression (LR) analysis. Cases with a ‘maybe’ response were removed from the analysis to compare those that were planning to leave (yes) and those that were definitely planning to stay (no). Backwards stepwise elimination was used to give us a more parsimonious model. A grouped scatter plotted the proportion of vacancies, additional cases and missed care. We plotted the observed groups and predicted probabilities. Data were aggregated across all four countries. We did not carry out a subgroup analysis by country due to the comparatively smaller sample size in 3 out of 4 countries.

We used the first 4 digits of work postcode as provided by participants to identify participants that may have been on the same team. We did not identify any participants with the same postcode and did not exclude any participants on this basis.

A visual inspection of data for outliers was carried out. All data was inspected for unusual values. All data were deemed to be reasonable within the data set.

## Results

### Participants

A total of 533 respondents completed at least 90% of the survey. Out of the 533 participants, the majority were from England (*n* = 357, 67%), followed by Scotland (*n* = 70, 13%), Northern Ireland (*n* = 51, 9.5%) and Wales (*n* = 39, 7.5%) and 16 (3%) did not provide sufficient postal/zip code information to determine the country. The majority of respondents were female (97.2%, *N* = 522).

Half of the respondents (51%, *n* = 269) reported that they held the specialist district nursing qualification (SPQDN) recognised in the UK. Registered nurses in the UK who work in the community identify in a number of ways. Almost half, 48.2% (*n* = 254) identified as *district nurse,* 41.5% (*n* = 219) as *community staff nurse*, 5.8% (*n* = 30) as *nurse specialist* and 4.5% (*n* = 24) as *community matron* (missing values *n* = 6).

The distribution between pay grades was 34% (*n* = 184) Afc Band 5, 41% (*n* = 221) Afc Band 6, 21.6% (*n* = 116) Afc Band 7 and 12 (2.2%) in Afc Band 8 *(missing cases, n = 6.)* There was a strong association between career progression (in terms of banding level) and whether the respondents had an SPQDN qualification. The majority of RNs at band 5 (98.4%) lacked an SPQDN qualification, and the majority of band 8 RNs had an SPQDN qualification.

In our sample, 45.5% (*N* = 240) reported that they were satisfied with their job, whilst 54.5% (*n* = 288) reported that they were dissatisfied. Among these, 27.6% said that they intend to leave their job, 35.6% that they might leave their current job and 37.5% that they will remain in their current job (see Table 1). Our findings indicate that there is nearly one-third of respondents who are undecided about whether they intend to leave their job. This implies that there is a dissatisfaction and if conditions worsen there will potentially be a much lower retention than what the current figures indicate.

**Table 1:** Intention to Leave and Job Satisfaction (*n* = 528)

		Intend to Leave the current Job			Total
		Yes	Maybe	No	
Are you satisfied with your job	Yes	25 (17.1%)	71 (38.2%)	144 (73.5%)	240 (45.5%)
	No	121 (82.9%)	115 (61.8%)	52 (26.5%)	288 (54.5%)
Total		146 (27.6%)	186 (35.6%)	196 (37.5%)	528

The average team size reported was 12.34 ± 9.6 RNs per team. Respondents were asked to indicate how many vacancies currently existed within their team. In our sample, 89.6% of all teams reported that they did not have a full complement of permanent staff on their last shift.

On average, respondents reported 9.2 ± 1.96 hours worked on their last shift. In community settings shifts vary in length from between 8 to 12 hours. Therefore, we have only asked about the length of their last shift and how much overtime they worked.

In terms of overtime work, respondents were asked how many minutes of overtime they had worked on their last shift. In the sample, 78% (*N* = 415) of respondents had done some overtime on their last shift. Out of the 415 respondents, who listed that they had worked overtime, the average overtime worked was 99.60 ± 58.25 minutes. Respondents were asked about management support received, 54.4% reported that they were not receiving sufficient support from their manager or management (see Table 2).

**Table 2:** Descriptive Analysis

**Individual and Organisational Outcomes**	**N (%)**
Did you Miss Care? (Yes)	319 (59.4%)
Do you Receive Sufficient Support from your Manager? (No)	235 (54.4%)
Have your working conditions worsened in the last year? (Yes)	448 (84.8%)
Did the Shift have a full complement of staff? (No)	476 (89.5%)
**Scalar**	**Mean, SD**
Years of Nursing Experience	13.7 ± 9.8
Active Caseload	205 ± 204
Team Size (RNs only)	12.3 ± 9.6
Permanent Staff Proportion	79.9% ± 18.6
Length of Shift (hours)	9.2 ± 1.96
Hours of Overtime	0.55 ± 0.7
Additional Cases (on last shift)	4.9 ± 10.7
N of Patients Seen (on last shift)	11.4 ± 9.7
COVID-related cases	20.7 ± 56 (*n* = 405, 77%)
Travel Mileage (on last shift)	27.9 ± 22.4

### Additional caseload

The respondents were asked about the number of additional cases that they had to take on their last shift due to staff absence. The average number of additional patients per shift per RNs due to staff absence was 4.9 ± 10.7. There were regional variations. The highest additional patients per RNs were reported in England (5.2 ± 12.6), followed by Scotland (4.9 ± 5.9), Wales (3.8 ± 4.1) and Northern Ireland (2.7 ± 2.9).

Respondents were also required to indicate increases in caseload because of COVID-19 pressures. More than three-quarters (*n* = 405, 77%) of RNs reported that their caseload had increased because of COVID-related cases. The burden of COVID-related cases was 20.7 ± 56. In England, average COVID-cases per RN were reported as 24.9 ± 66.1, which is nearly a 30% increase in case load due to COVID-related cases. In Northern Ireland, average additional cases per RN were 14.4 ± 33.3 (20% of total cases load), in Wales 9.3 ± 11.7 (20% of total case load) and in Scotland 7 ± 11.8 (8% of total case load).

There is no assumption in this table of normality due to using Non-parametric tests (chi-squared and Kruskal-Wallis), therefore outliers have a marginal effect on the results. There were no extreme outliers identified. Extreme outliers were defined as those that were unlikely or impossible (eg. 1000 hours etc.). *Manager Support, team size, permanent staff proportion, length of shift, hours of overtime, working conditions in the last year, job satisfaction, overall working conditions* and *travel mileage* were significantly different between the *yes, no and uncertain intention to leave* outcomes. *Conditions worsened in the last year* variable, although borderline to significance (*p* = 0.08), was included because it was deemed important as it indicates whether conditions and dissatisfaction have worsened due to the COVID-19 pandemic (see Table 3).

**Table 3:** List of variables and factors to be included in predicting DNs intention to leave- Kruskal Wallis and Chi square tests

Dichotomous	Intention to Leave				
	Yes	No	Maybe	Test	*p*-value
SPQDN Qualification (Yes)	48.6%	52.7%	51%	Chi-squared (2) =0.5	0.764
Did you Miss Care (Yes)	67.1%	59.1%	56.1%	Chi-squared (2) =4	0.113
Do you Receive Sufficient Support from your Manager (No)	81.0%	55.6%	31.4%	Chi-squared (1) =69	< 0.01
Have your working conditions worsened in the last year (Yes)	92.5%	83.9%	80.1%	Chi-squared (2) =10	0.06
Are you Satisfied with your Job (No)	82.9%	61.8%	26.5%	Chi-squared (2) =113	< 0.01
Did the Shift have a full complement of staff (No, *N* = 472)	91.1%	87.2%	90.3%	Chi-squared (2) =1.57	0.456
**Scalar**					
Years of Nursing Experience	14.3 ± 11	13.1 ± 9.8	13.8 ± 8.5	H (2) =2.3	0.319
Team Size	11 ± 10	12.7 ± 8.5	13 ± 10.4	Kruskal-Wallis H (2) =7.9	< 0.05
Permanent Staff Proportion	74.6% ±20.5	83% ± 20	80.8%17.2	Kruskal-Wallis H (2) =18	< 0.001
Length of Shift	9.4 ± 2.2	9 ± 1.9	9.2 ± 1.7	Kruskal-Wallis H (2) =9.8	< 0.01
Hours of Overtime	1.4 ± 1.1	1.05 ± 0.99	1.3 ± 0.98	Kruskal-Wallis H (2) =12.2	< 0.01
Caseload Current Active	208 ± 195	199.7 ± 195	202.7 ± 213.6	Kruskal-Wallis H (2) =0.8	0.669
Additional Cases (on last shift)	4 ± 4	4.2 ± 9.8	6.1 ± 14.5	Kruskal-Wallis H (2) =3.3	0.064
How would you rate your overall working conditions? (1 = Poor, 2 = Bad, 3 = Average, 4 = Good, 5 = Excellent)	2.4 ± 0.96	3.3 ± 0.79	2.8 ± 0.9	Kruskal-Wallis H92) =68.3	< 0.05
Travel Mileage (on last shift)	30.5 ± 22.8	25.1 ± 19.8	28.9 ± 24.5	Kruskal-Wallis H (2)=6	< 0.05

Significantly fewer people reported *job satisfaction* among those who were intending to leave than those who were uncertain, while fewer of those who were uncertain about leaving reported job satisfaction than those who intended to stay. Of those who intended to leave, 89% reported dissatisfaction with their job. This was significantly larger than those who were uncertain (61.8% were dissatisfied) and this was significantly larger again that those who indicated not intending to leave (26.5% were dissatisfied).

*Have your working conditions worsened in the last year* was significant and much the same pattern as job satisfaction but the proportion of those who say *maybe* is not significantly different from either % yes or % no. The percentage of those who indicated intention to leave who said that conditions had worsened (92%) was significantly higher than the percentage of those who indicated not intending to leave (80%). *Manager support* outcome was significantly different between all three intending to leave outcomes.

*Team size* was significantly lower between those that reported intention to leave compared to those who do not intend to leave. The difference between was not significant. *Permanent Staff Proportion* was significantly different between intention and not intending to leave (higher among those that intend to stay) and intending to leave and uncertain outcomes.

The *Overall working conditions* score outcome was analysed as a total mean score, Mean, SD. The score is significantly different between all three outcomes. Working conditions are rated lowest among those that intend to leave and highest among those that indicated not intending to leave. We also explored *number of patients seen* as an indicator of workload. Due to the significantly smaller response rate to this question (50%), we have not included it in the LR analysis. However, it is notable that despite a small sample size the differences are significantly different between those that Intend to leave, uncertain and not intending to leave. The number of patients seen is lowest among those not intending to leave (9.9 ± 6.4) and highest among those that indicate an intention to leave (13.5 ± 10.7), Kruskal-Wallis H (2) =11.8p = 0.03.

Likelihood of Multicollinearity was assessed by exploring correlations of independent variables prior to carrying out backwards LR. The ones that were highly correlated were chosen. The following terms were added to the carrying out the backwards LR.

### Logistic regression

This analysis was conducted on 527 respondents. Respondents were removed from the analysis because they were undecided (answered *maybe* to the Intention to leave question). Excluded from the analysis were all non-significant variables from Table 2.

Further, *Job satisfaction* was excluded because this variable is strongly correlated with the Intention to Leave outcome (80% of those that are dissatisfied are considering leaving or are leaving compared with 17.1% of those who were satisfied). In addition, job satisfaction is an umbrella term that covers many of the reasons for leaving employment in a number of industries and therefore was seen as effectively measuring the same thing with a slightly different question.

Also excluded was the *number of patients* variable despite being significant as there was a substantially lower response rate to this question (*n* = 314, 58.5%). This would have decreased the power of the analysis substantially and thus it was felt wise to withdraw this variable from the analysis.

Included in the LR were *management support, team size, shift length, overtime, travel mileage, overall self-rated working conditions and worsened conditions in the last year*. Although *worsened conditions in the last year* variable was borderline significant it was included in the analysis because it is indicating whether intention to leave is impacted by the COVID-19 pandemic.

The model predicted 73.6% of cases of whether they intended to leave correctly. The model was significant Df (4) 92.474, *p* < 0.01. Overtime, manager support, proportion of permanent staff and self-rated overall working conditions were significant in the model. Respondents that did more overtime were more likely to indicate that an intention to leave. For each hour of overtime, they were approximately 40% more likely to indicate an intention to leave. Participants reporting that they did not receive manager support were more likely to report intention to leave. The lower proportion of permanent staff on the team the more likely participants are to say they intend to leave. Also, the lower the overall self-rated working conditions, the higher the intention to leave (see Table 4).

**Table 4:** Logistic Regression- the independent variables selected in the final model as contributing to the Prediction of Intention to Leave (Yes, No)

Variable	B	s.e.	p-value	Odds Ratio	CI
Overtime	.299	.156	.056	1.349	0.992–1.833
Do you receive sufficient support by your Manager?	−1.829	.330	< 0.001	0.161	0.084–0.307
Proportion of Permanent Staff	−.021	.008	.009	0.980	0.965–0.995
(1 = Poor 5 = Excellent) - Working Conditions	−.720	.185	< 0.001	0.487	0.339–0.699

The LR analysis results in Table 4 indicate that the odds of intending to leave increase by 30% for every hour of overtime reported. Respondents who felt supported by their managers has 83.9% lower odds for intending to leave. An increase in positions filled of 20% will result in a 34% drop in the odds ratio of someone intending to leave. For someone rating their working conditions as excellent, the odds of intending to leave is 94% lower than someone who rates their working conditions as poor. Overtime is only significant on the 10% level. The independent variable selected in the final model is contributing to the prediction of the intention to leave.

## Discussion

For some time now, UK commentators have identified the challenges being experienced within the community nursing workforce [25]. These UK concerns are reflective of the changes occurring on a global scale. Our findings indicate that the proportion of community nurses in our survey who have indicated an intention to leave is high, which is of serious concern when considering the future of community-based health care provision. However, the prevalence of Intention to Leave anticipation is not exceptional and is consistent within a wider international literature. Delobelle et al. indicate similar levels of intention to leave in a South African community context [26]. Similar rates of intention to leave are also noted in a study of Public health nurses in Canada and community nurses in Saudi Arabia [27, 28]. To the best of our knowledge, this is the first UK study to provide robust self-report data indicating such elevated levels of intention to leave. Our findings showed that excessive overtime, reduced proportion of permanent staff, poor management and support and overall working conditions are the four factors that have the highest impact on the community nurses who answered that they intend to leave, compared to those that answered that they are happy staying in their job. It is important to note here that patient numbers as a variable was removed from the final LR. However, it is our understanding that other variables (notable overtime and reduced proportion of staff) may act as proxies.

Studies focusing on retention intervention strategies are numerous. Although reviews on the subject tend to be exclusively focused on the acute nursing workforce, it is worth noting here that evidence supporting enhanced mentorship, increased access to supervision, team cohesion and orientations, interventions and leadership behaviour interventions, are seen to improve retention [18, 29]. In a community context Chamanga et al., point to enhanced teamwork, access to education, improving work-life balance and appreciation by managers, as factors that increased retention for nurses [30]. Whilst these interventions show some promise and do begin to address some of the concerns expressed by nurses in our study, the quality of the evidence is relatively poor. Furthermore, those types of retention interventions might be described as ‘missing the point’ somewhat, in failing to tackle the very serious material concerns that many participants had. In other words, only part of our underlying conceptual model is addressed by such interventions.

These material or economic aspects of the model are relevant in terms of workforce resource. Expectations to frequently work overtime is a result of too high a workload. It is notable that *longer shifts, inadequate staffing levels and poor support* have previously been identified as a predictor of nurse burnout [31]. Our findings also suggest that a low proportion of permanent staff within the team is a determinant of staff intending to leave. Working with permanently understaffed teams puts more strain on other staff and is therefore an expected indicator of intention to leave. We know from our own work on ‘missed care’ that the proportion of temporary staff in community nursing teams is a factor in determining quality of care [32]. Whilst we know that self-reported quality of care delivered is a factor in job satisfaction [32] it stands to reason that this underlying team material factor would also determine nurses intention to leave. Staff wellbeing and staff burnout have resulted in missed and/or delayed patient care, which was reported by all our participants but was highest among those that intend to leave (67.1%). The effect of burnout has been reported to have inevitable effects on patients. Outcomes from burnout include: reduced job performance, poor quality of care, poor patient safety, adverse events, patient negative experience, medication errors, infections, patient falls, and intention to leave [31]. This previously evidenced relationship explains why being able to provide good care is of central importance and contributes to the conditions that would help participants in our study be more satisfied at work, achieve manageable workloads and reduce burden.

Previous studies have demonstrated that high workload and burnout are factors that influence nurses’ intention to leave [19]. These other studies note reduced quality of care as a consequence of burnout, we add that observing poor quality of care and not achieving this, is in itself a contributing factor to burnout, and explains why being able to provide good care is of central importance and contributes to the conditions that would help participants in our study be more satisfied at work, achieve manageable workloads and reduce burden.

Our finding that enhanced levels of overtime in community settings are a factor in intention to leave is therefore not surprising. Likewise, managerial style and perceived lack of managerial support are also cited as sources of dissatisfaction leading to risk of nurses leaving the profession. In particular, management by exception and the presence of transactional leadership styles are reported as factors that increase intention to leave rates [33]. Importantly, this work further highlights the significance of the relationship between organisational and resource factors, alongside labour supply and other wider sets of conditions as being critical to the retention of community and district nurses [22].

To address the economic and material aspects of workforce retention, policy initiatives should focus on ensuring that community nursing services have sufficient resources and funding to be able to employ a safe and required number of staff at appropriate grades. This will ensure that staff workload is manageable, and that the nursing profession is able to deliver the quality of care that they and patients are satisfied with. For too long the issue of retention within the community nursing workforce has been ignored, a problem exacerbated with policy changes within the Health and Social Care act [34]. Despite an already growing gap in the clinical workforce, and worsening recruitment and retention, the focus by NHS England became prevention, integration and technology with no mention or focus on retention [35]. Kuhlman et al., summarise what is required for effective primary and community health care to develop in a way which meets the challenges of the twenty-first century [3]. They argue that large scale institutional change, political commitment and the required resources are needed to help deliver ‘people-centred’ primary and community health care provision. The retention issue highlighted here, sits at the very heart of these challenges and we concur that leadership at a national level is desperately needed to address the complex cultural and material challenges.

Conclusions: This study of 533 community nurses from across the UK has provided important insights into the work experiences of those intending to leave their job. The patterns identified by this study consistently shows that adverse job characteristics - high workload, low staffing levels, long shifts, low control - are associated with burnout in nursing. The potential consequences for staff and patients are severe.

Those intending to leave their job reported lower job satisfaction, worsening working conditions, lower proportion of permanent staff, poorer working conditions, and smaller team size than those who intended to stay. These findings have important implication for policy makers, NHS managers, nurse leaders and nurses in addressing nursing workforce retention in community settings. A collapse of care provision in community settings in inevitable, if poor management, as currently causing low morale does not change.

Our evidence would suggest that there exists an organisational culture which does not recognise that ‘people-centred’ also means a recognition of the well-being needs of health care professionals within the primary health care system and given their central role, especially nurses.

### Limitations

Despite a relatively large sample size, the participants in the study represent only a small fraction of the overall UK community nursing workforce. The participants are self-selecting, and this may have led to a form of bias. The data is exclusively comprised of self-report, with potential for recall bias.

## Data Availability

The data that support the findings of this study are available upon request, but restrictions apply to the availability of these data, which were used after obtaining Ethical Approval for the current study, and so are not publicly available. For any data queries please contact the lead author.
